# Associated factors of depression, anxiety, and suicide behavior among men in Switzerland: findings from the Swiss health survey 2022

**DOI:** 10.3389/fpsyg.2026.1725181

**Published:** 2026-02-04

**Authors:** Nora M. Laskowski, Roland Müller, Markus Theunert, Georgios Paslakis

**Affiliations:** 1 University Clinic for Psychosomatic Medicine and Psychotherapy, Medical Faculty, Campus East-Westphalia, Ruhr-University Bochum, Luebbecke, Germany; 2 Männer.ch, Competence Centre for Men’s Health, Bern, Switzerland

**Keywords:** anxiety, depression, men’s mental health, population-based study, suicidality, Swiss health survey

## Abstract

This study examined demographic, socioeconomic, and psychosocial factors associated with symptoms of depression, anxiety, and suicide attempts among men in Switzerland, using nationally representative population data from the Swiss Health Survey 2022. Understanding men’s mental health disparities is essential, as men are often underdiagnosed and less likely to seek psychological help despite high suicide rates. Data from 10,761 individuals registered as male were analyzed. All analyses applied the survey weights provided by the SFO to ensure representativeness of approximately 3.55 million adult men living in private households across all Swiss regions. Depressive symptoms were assessed using the Patient Health Questionnaire-9 (PHQ-9) and anxiety symptoms with the Generalized Anxiety Disorder-7 (GAD-7). Lifetime suicide attempts were assessed through self-report. Weighted linear and logistic regression models examined associations between mental health outcomes and demographic (age, language area, partnership status), socioeconomic (income, employment, education), and migration-related factors, including sexual orientation. Higher symptom levels were associated with younger age, lower income, unemployment, and single relationship status. Men identifying as non-heterosexual and those with a migration background reported significantly higher scores on both the PHQ-9 and GAD-7 compared to heterosexual and Swiss-born men. Regional and language differences were evident, with elevated symptom levels among French-speaking participants. Transgender and non-binary individuals registered as male exhibited particularly elevated symptom levels of anxiety and depression. These findings highlight persistent disparities in men’s mental health across social and demographic groups in Switzerland. Population-level screening, improved access to gender-sensitive mental health services, and targeted prevention programs addressing social and economic vulnerability are warranted. Future national surveys should also incorporate measures of masculinity norms and gender role attitudes to better capture underlying mechanisms contributing to men’s mental health outcomes.

## 1 Introduction

Male mental health has historically received insufficient attention in healthcare, policy, and research (Bilsker et al., 2018). Despite increasing awareness, it remains a critical yet frequently overlooked public health issue (Möller-Leimkühler, 2002; McKenzie et al., 2018; Rice et al., 2018; Walther, 2025). Epidemiological studies consistently report lower prevalence rates of depression (Salk et al., 2017) and anxiety (Watterson et al., 2017) among men compared to women. At the same time, men account for a disproportionate share of suicide deaths, highlighting a persistent gender paradox in mental health outcomes (Bilsker et al., 2018; Hedegaard et al., 2018; Rice et al., 2024). In many Western countries, suicide rates among men can be up to seven times higher than among women (Bilsker et al., 2018). Untreated psychological conditions in men are associated with substantial functional impairment, comorbidity, and premature mortality—often occurring during the most economically and socially productive phases of life (Möller-Leimkühler, 2002).

Mental health outcomes are shaped by an interplay of biological, psychological, and social factors. Beyond genetic vulnerability and physiological mechanisms, psychosocial environments and gendered expectations strongly influence how psychological stress develops, is perceived, and is expressed (Kirkbride et al., 2024). Gender identity and gender roles play a central role in this process, as individuals often enact and internalize culturally defined norms of masculinity and femininity that shape emotional regulation, coping strategies, and health-related behaviors (Hammarström et al., 2014). In men, gender role conflict—the psychological strain resulting from rigid, restrictive, or contradictory male role expectations—has been associated with poorer mental health outcomes and emotional dysfunction (Möller-Leimkühler, 2002; Seidler et al., 2016; Rice et al., 2018; Walther, 2025). Similarly, traditional masculinity norms, such as self-reliance, emotional restraint, and aversion to vulnerability, have been identified as key barriers to recognizing and communicating distress (Tedstone Doherty and Kartalova-O’Doherty, 2010; Rice et al., 2018).

Beyond individual-level norms and role expectations, this line of research has been extended by scholarship on masculinities, conceptualizing masculinities as relational and hierarchical social processes, including multiple coexisting masculinities, with some forms culturally privileged over others (Connell and Messerschmidt, 2005; Messerschmidt, 2019). In this framework, *hegemonic masculinity* refers to historically and contextually embedded patterns of masculinity that perpetuate unequal gender relations, defined in relation to other (non-hegemonic) masculinities and femininities rather than as a fixed set of traits (Connell and Messerschmidt, 2005). The same framework explicitly includes *subordinated* and *marginalized masculinities—*i.e., masculinities positioned lower in the alleged hierarchy and/or devalued through intersections with, e.g., sexuality, class, ethnicity, or age (Connell and Messerschmidt, 2005; Messerschmidt, 2019). Conceptualizing men’s mental health through this lens emphasizes that distress and coping are shaped by the social consequences of proximity to, or divergence from hegemonic masculine ideals (Connell and Messerschmidt, 2005; Messerschmidt, 2019).

These gendered processes are closely intertwined with stigma surrounding mental illness. Many men fear that acknowledging psychological distress will be perceived as weakness, potentially conflicting with societal ideals of masculinity (Seidler et al., 2016). Such stigma not only affects help-seeking behavior (Möller-Leimkühler, 2002) but may also shape symptom expression and reporting. Emerging evidence suggests that male-typical manifestations of distress—such as irritability, risk-taking, or substance use—are less likely to be recognized as indicators of depression or anxiety within clinical and diagnostic frameworks (Rice et al., 2018; Walther, 2025). As a result, mental health problems among men may be underdiagnosed and undertreated, and prevalence estimates based on self-report or service contact may underestimate the actual burden of mental illness in male populations.

Importantly, men do not represent a homogeneous group. Intersectionality provides a critical framework for understanding heterogeneity in men’s mental health by emphasizing how gender interacts with other social categories, including sexual orientation, ethnicity, socioeconomic status, and migration background (Hammarström et al., 2014; Opozda et al., 2024). For instance, individuals with non-heterosexual orientations experience disproportionately high rates of depressive symptoms and generalized anxiety disorder (GAD) (Wang et al., 2007a, 2007b, 2014; Chakraborty et al., 2011; Malik et al., 2023). Social exclusion, discrimination, and minority stress have been identified as key mechanisms driving these disparities (Courtenay, 2000). Evidence from Swiss and international studies consistently demonstrate an elevated mental health burden among sexual minority men, underscoring the need for population-based analyses that capture within-group diversity in men (Wang et al., 2007a, 2014).

From a masculinities perspective, these intersectional disparities may be understood as reflecting differential social positioning within hierarchies of masculinity. Men who diverge from culturally dominant, hetero- and cis-normative masculine ideals—such as sexual minority men or gender-diverse individuals registered as male—may be positioned in subordinated (marginalized) masculinity categories, which are associated with reduced social legitimacy and increased exposure to chronic stressors (Connell and Messerschmidt, 2005; Messerschmidt, 2019). Such positioning may shape the vulnerability to mental health problems and the ways in which distress is expressed, recognized, and responded to within healthcare systems and broader social contexts.

At the same time, masculinities scholarship cautions against conceptualizing masculinity exclusively in terms of risk or deficit. Strength-based perspectives emphasize that certain practices that are traditionally thought of as masculine—such as responsibility for others, action-oriented forms of care, and (functional) forms of self-reliance—may function as psychological resources, if they are flexibly enacted and socially supported (Kiselica and Englar-Carlson, 2010). Incorporating such perspectives allows for a more differentiated understanding of men’s mental health, acknowledging that masculinities can operate both as a source of vulnerability and as a potential resource depending on context, rigidity of expression, and social valuation.

Addressing men’s mental health therefore requires an integrated public health perspective that considers social determinants, structural inequities, and the cultural context in addition to individual-level risk factors (Brooks, 2001; Rice et al., 2024). This perspective is particularly relevant in Switzerland, a linguistically and culturally diverse country with four national languages and pronounced regional identities. Specific cultural contexts may shape exposure to stressors, norms of emotional expression, and access to care. Prior research has demonstrated regional differences in physical and mental health outcomes across Swiss language areas (Schulz et al., 2013; Roser et al., 2019), highlighting the importance of considering the regional context in population-based mental health research.

Although several studies based on the Swiss Health Survey (SHS) have examined mental health-related outcomes, most have focused on specific exposures or subdomains, such as work-related stress (Scholz-Odermatt and Zyska Cherix, 2024), or unemployment and over-indebtedness (Hämmig, 2024), or mental health service utilization (Dey and Jorm, 2017), often without gender as a central analytic variable. Even when gender is included, research typically emphasizes comparisons between men and women—for example, in disordered eating (Forrester-Knauss and Zemp Stutz, 2012) or body dissatisfaction (Richard et al., 2016)—rather than examining heterogeneity within male populations. To date, no population-based study in Switzerland has systematically investigated men’s mental health across intersecting dimensions such as gender identity, sexual orientation, socioeconomic factors, and regional or linguistic background using nationally representative data.

The present study addresses this gap by analyzing male respondents from the most recent SHS (2022). Specifically, we aim to (1) estimate the prevalence of anxiety, depression, and suicide attempts among men in Switzerland and (2) identify sociodemographic, sexual orientation, and regional factors associated with these outcomes. By explicitly focusing on within-group diversity among men—including cisgender, transgender, and non-binary individuals registered as male—this study extends existing Swiss mental health research beyond binary gender comparisons. In doing so, it provides novel, population-based evidence on intersectional and regional profiles within male populations and contributes to a more nuanced understanding of men’s mental health in the Swiss public health context. The findings are intended to inform public health policy and guide the development of prevention and intervention strategies tailored to the needs of diverse male populations.

## 2 Materials and methods

### 2.1 Data source

Data were drawn from the SHS 2022 [Bundesamt für Statistik (BFS), 2023], a nationally representative, population-based survey conducted by the Swiss Federal Statistical Office (FSO). The SHS follows a cross-sectional design and is administered every 5 years to assess health-related behaviors, chronic conditions, and psychosocial well-being in the resident population of Switzerland. The survey uses a stratified random sampling procedure to ensure representativeness across all linguistic regions and major demographic strata.

Data collection was carried out via computer-assisted telephone interviews (CATI) followed by a self-administered online or paper questionnaire. The FSO applies post-stratification weights based on age, sex, nationality, and region to correct for differential response probabilities and ensure that estimates reflect the adult population living in private households in Switzerland. Detailed documentation on survey design, quality control, and data reliability is available from the FSO [Bundesamt für Statistik (BFS), 2023].

The psychometric quality of key measures within the SHS has been evaluated in previous cycles, demonstrating acceptable internal consistency and criterion validity for large-scale population use [Roser et al., 2019; Bundesamt für Statistik (BFS), 2023].

### 2.2 Participants

The present analysis included 10,761 individuals who were registered as male in civil status records, aged 15 years or older, and residing in Switzerland at the time of the survey. Participants were included regardless of their current gender identity to capture within-group variability among all individuals registered as male. Cases with missing data on key variables (depression, anxiety, or suicide attempt) were excluded from analyses.

To ensure population-level representativeness, all analyses applied the official survey weights provided by the FSO [Bundesamt für Statistik (BFS), 2023]. After weighting, the analytic sample corresponded to an estimated 3.55 million adult men living in private households across Switzerland.

Demographic variables included age, educational attainment, employment status, income, partnership status, language region (German, French, or Italian), migration background (Swiss-born vs. foreign-born), and sexual orientation (heterosexual vs. non-heterosexual).

### 2.3 Measures

For the purpose of statistical analyses, anxiety and depressive symptoms (Generalized Anxiety Disorder-7 [GAD-7] and Patient Health Questionnaire-9 [PHQ-9] sum scores) were treated as continuous variables. Lifetime suicide attempt was modeled as a dichotomous variable. Sociodemographic variables comprised a combination of continuous (age, employment rate, net monthly household income, number of persons in household), ordinal (educational attainment), and categorical variables (gender identity, sexual orientation, employment status, partnership status, migration status, nationality, residential area, and language region). Categorical variables were dummy-coded as appropriate for inclusion in the regression models.

#### 2.3.1 Anxiety symptoms

Anxiety symptoms were assessed with the GAD-7 (Spitzer et al., 2006), which measures the frequency of seven anxiety-related symptoms over the past 2 weeks on a 4-point Likert scale (0 = not at all to 3 = nearly every day). Total scores range from 0 to 21, with higher scores representing greater symptom burden. Following established interpretative cut-offs, scores of 10 or above indicate moderate or higher anxiety levels. The GAD-7 has demonstrated excellent reliability and validity in community samples, and internal consistency in the SHS 2022 data was high (Cronbach’s *α* = 0.89).

#### 2.3.2 Depressive symptoms

Depressive symptoms were assessed using the PHQ (Kroenke et al., 2001), a widely used screening instrument for depression in population-based surveys. The PHQ-9 measures the frequency of nine core depressive symptoms during the past 2 weeks on a 4-point scale (0 = not at all to 3 = nearly every day), yielding total scores from 0 to 27. Higher scores indicate greater symptom severity. Scores ≥ 10 reflect at least moderate depressive symptomatology. In the SHS 2022, the PHQ-9 demonstrated high internal consistency (Cronbach’s α = 0.86) and strong construct validity across previous SHS waves [Bundesamt für Statistik (BFS), 2023].

#### 2.3.3 Suicidal behavior

Suicidal behavior was assessed through a standardized item from the SHS self-completion questionnaire: “Have you ever attempted to take your own life?” (yes / no). Responses were coded dichotomously (1 = yes, 0 = no). This question corresponds to the suicide-attempt item used in the World Health Organization (WHO) World Mental Health Surveys (Nock et al., 2008) and provides a validated, ethically appropriate indicator of lifetime suicide attempts in population-based research. While it does not assess suicidal ideation or planning, it captures the key behavioral component of suicidal acts as defined in epidemiologic research.

In the SHS, this assessment included follow-up questions for respondents reporting a suicide attempt, asking whether they had discussed the attempt with (1) someone in their private social network, (2) a physician or other healthcare professional, (3) a counselor or institution, or (4) no one. Each of these items was answered with the response options applies or does not apply.

All instruments were administered as part of the standardized self-completion module and underwent translation and back-translation into German, French, and Italian according to FSO protocols to ensure linguistic and conceptual equivalence.

### 2.4 Sampling and procedures

The SHS has been conducted every 5 years since 1992 by the FSO. The sampling framework is based on cantonal and municipal resident registers, which are updated quarterly with information from telephone service providers. A stratified random sampling design is employed, with the cantons serving as strata. The sample allocation ensures a minimum sample size for all major regions. The target population includes individuals aged 15 years and older living in private households, encompassing foreign nationals who have held a residence or short-stay permit for at least 12 months.

In 2022, the national sample included 60,651 individuals, of whom a net sample of 10,000 was designated for national estimates. Several cantons and the city of Zurich opted for sample extensions to support regional analyses, adding 1,000 additional telephone interviews with foreign nationals. In total, 21,930 telephone interviews were completed, yielding a response rate of 36.2% [Bundesamt für Statistik (BFS), 2023].

The SHS uses a mixed-mode design to optimize participation. Most respondents completed CATI lasting approximately 37 min. For participants unable to participate by phone, computer-assisted face-to-face interviews (CAPI) were offered (*n* = 3). Proxy interviews were conducted when health or language barriers prevented direct participation (*n* = 686; 3%). After the interview, respondents were invited to complete a self-administered questionnaire, either online (74%) or on paper (26%), resulting in 19,137 returned questionnaires and a response rate of 90.1%. Interviews were conducted in German, French, and Italian between January 17 and December 22, 2022, with data collection evenly distributed throughout the year to minimize seasonal bias [Bundesamt für Statistik (BFS), 2023].

Data quality was ensured through multiple procedures [Bundesamt für Statistik (BFS), 2023]. Real-time plausibility checks were embedded within the CATI system, and manual consistency checks were performed during data cleaning. Demographic variables (e.g., age, gender, household composition) were verified through linkage with register data. The final dataset was validated for completeness, internal consistency, and accuracy before release.

All procedures complied with Swiss data protection laws and the Declaration of Helsinki. Participation was voluntary, and all data were fully anonymized prior to analysis. Informed consent was obtained from all participants. Ethical oversight and approval were provided by the FSO, which serves as the data owner and coordinating body for the SHS.

### 2.5 Analyses

#### 2.5.1 Weighting

Data from the SHS 2022 are representative of the Swiss population aged 15 years and older living in private households [Bundesamt für Statistik (BFS), 2023]. To obtain accurate population estimates, survey weights provided by the FSO were applied to correct for the sampling design, differential response probabilities, and demographic composition. After weighting, the dataset represented 7,182,252 individuals, corresponding to the total resident population of Switzerland.

For the present analyses, only individuals recorded as male were included (*N* = 3,549,209), encompassing cisgender men, transgender men, and individuals registered as male in civil status records who identified as non-binary or with an unspecified gender identity.

The weighting procedure consisted of three main steps [Bundesamt für Statistik (BFS), 2023]. First, design weights were calculated to correct for unequal selection probabilities across cantons. Second, nonresponse adjustments were applied using model-based weights that accounted for predictors of participation. Finally, calibration weights aligned the sample distributions with known population parameters, including region, age, gender, nationality, marital status, and household size. This multistage process corrected for regional disproportionalities and ensured that the weighted data accurately reflected the demographic structure of the Swiss resident population.

#### 2.5.2 Statistical analysis

The analyses were conducted using IBM SPSS Statistics (IBM Corp, 2022) version 30.0.0.0 and Microsoft Excel version 2,108 (Microsoft, 2025). Figures were created using Python version 3.12.4 (Python Software Foundation, 2024) in a Jupyter environment (Jupyter Team, 2015) with matplotlib version 3.9.0 (Hunter, 2007) and pandas version 2.2.2 (McKinney, 2010).

All analyses were conducted using weighted data, applying the official survey weights provided by the FSO to ensure representativeness of the Swiss adult male population. Descriptive analyses included absolute (*n*) and relative frequencies (%), as well as means (*M*), standard deviations (SD), and ranges, where applicable.

To identify associated factors of depressive and anxiety symptoms, multiple linear regression analyses were conducted using continuous PHQ-9 and GAD-7 scores as dependent variables. Categorical factors with more than two levels were dummy-coded. Regression results are presented as unstandardized coefficients (*B*), standard errors (SE), standardized coefficients (*β*), *t*-values, *p*-values, and 95% confidence intervals (CIs). Multicollinearity among associated factors was assessed using variance inflation factors (VIFs). All VIF values were well below conventional thresholds (all VIFs < 2), indicating no multicollinearity concerns.

For suicidal behavior, a binary logistic regression was performed with lifetime suicide attempt (yes/no) as the dependent variable. Results are reported as *B*, SE, Wald statistics, *p*-values, exponentiated coefficients (*Exp[B]*), and 95% CIs. Odds ratios (ORs) were used to compare the relative strength of each factor.

## 3 Results

### 3.1 Sample characteristic

For the present analyses, data from the telephone interviews and self-administered questionnaires of the SHS 2022 were merged, including only respondents who participated in both components. All analyses were conducted using the weighted data as described in Section 2.5.

The analytic sample included all individuals recorded as male in civil status records (*N* = 3,549,209, weighted). This population consisted of:

1. Cisgender men (99.33%; *n* = 3,481,960),
2. Transgender men, i.e., individuals assigned female at birth who now identify as male (0.50%; *n* = 17,448), and
3. Individuals registered as male but identifying with a non-binary or unspecified gender identity (0.17%; *n* = 6,392).

In addition, 43,408 respondents did not report their current gender identity but were classified as male according to the civil status register and therefore included in the analysis. Individuals with a female gender identity were excluded.

This inclusive approach allowed for a comprehensive examination of the diversity within the male population, reflecting both cisgender and gender-diverse individuals recorded as male. To facilitate subgroup analyses, a categorical gender identity variable was created, distinguishing between cisgender, transgender, and non-binary/other male-registered participants. The sociodemographic characteristics of the weighted sample are presented in Table 1.

**Table 1:** Weighted sociodemographic characteristics of male participants in the Swiss health survey 2022.

Variable	Weighted descriptive statistics
Age	*M* (SD); Median [Range]
	48.02 (18.86); 48 [15–100]
Sexual orientation	*N*(%)
Heterosexual	3,112,577 (92.6)
Homosexual	100,299 (3.0)
Bisexual	55,610 (1.7)
Other, not specified	92,999 (2.8)
Marital status	
Single^1^	1,758,053 (49.5)
With partner^2^	1,791,157 (50.5)
Persons in household	
	*M* (*SD*); Median [Range]
	2.67 (1.36); 2 [1–12]
Nationality	
Swiss at birth	2,614,201 (73.7)
Non-Swiss	935,008 (26.3)
Migration status	
Without	2,182,199 (62.0)
First generation	1,038,549 (29.5)
≥2. Generation	300,999 (8.5)
Education	
Compulsory education (9 years incl. primary school)	468,187 (13.3)
Secondary school level II	1,483,875 (42.0)
Tertiary school	1,576,827 (44.7)
Employment status	
Non-employable person (e.g., pensioners)	944,035 (26.6)
Unemployed	78,470 (2.2)
Employed	2,526,317 (71.2)
Employment rate (%)	
	*M* (*SD*); Median [Range]
	89.89 (23.21); 100 [1–100]
Net monthly household income^3^	
	*M* (*SD*); Median [Range]
	8,576.06 (12,718.70)
	7,000 [0–400,000]
Residential area	
Urban	2,192,129 (61.8)
Intermediate (dense peri-urban area and rural zones)	788,709 (22.2)
Rural	568,371 (16.0)
Language areas	
German-speaking Switzerland	2,568,603 (72.4)
French-speaking Switzerland	836,550 (23.6)
Italian-speaking Switzerland	144,056 (4.1)

Values are based on weighted data representing the adult male population in Switzerland (*N* = 3,549,209). Missing data: sexual orientation (*n* = 187,724); migration status (*n* = 27,462); education (*n* = 20,320); employment status (*n* = 387); employment rate (*n* = 1,029,745); and net monthly household income (*n* = 2,048,634).

1 single = single, widowed, divorced, unmarried, dissolved registered partnership.

2 with partner = married, registered partnership.

3 in CHF.

### 3.2 Anxiety symptoms

Among men included in the weighted sample, 7.3% (*n* = 256,895; 5,866 missing responses) reported experiencing anxiety symptoms within the past 12 months. Of these, 145,721 respondents reported having received a medical diagnosis of GAD, corresponding to a weighted prevalence of 4.1%.

The mean GAD-7 score was *M* = 2.82 (SD = 3.41; 53,378 missing responses). Symptom-severity classification based on standard cut-off values indicated that 76.7% (*n* = 2,682,164) of men reported no symptoms, 18.1% (*n* = 637,939) reported mild symptoms, 3.5% (*n* = 123,916) reported moderate symptoms, and 1.5% (*n* = 51,813) reported severe symptoms.

For the association analyses, the GAD-7 total score was used as the dependent variable in a multiple linear regression model (see Supplementary Table S1). All sociodemographic variables examined were significantly associated with GAD symptoms.

**Figure 1:**
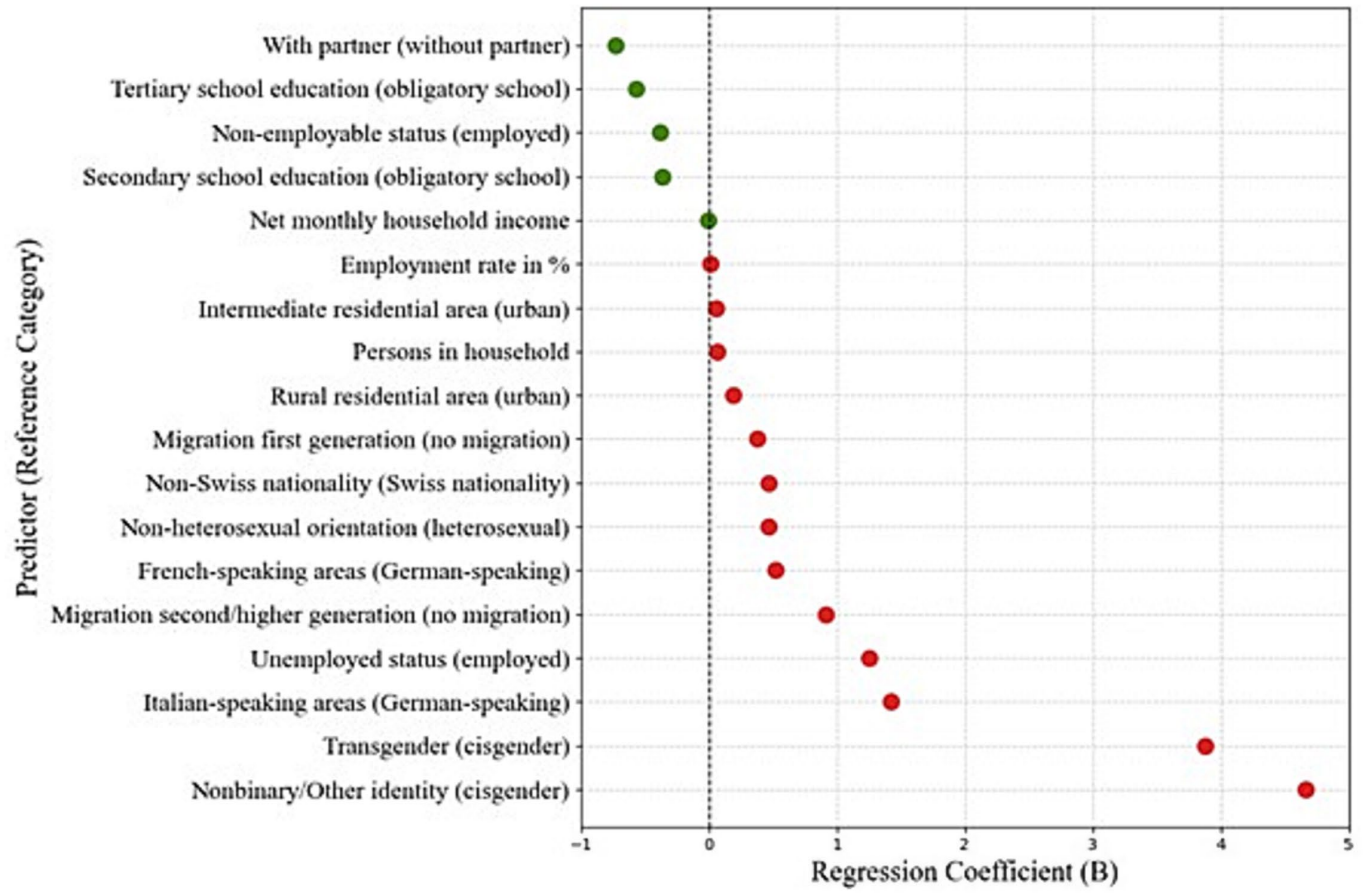
Associated factors of generalized anxiety disorder (GAD-7) based on regression coefficients. Green dots represent protective factors and red dots indicate factors associated with higher symptom levels. The 95% confidence intervals are not visible due to the narrow range.

As illustrated in Figure 1, gender identity emerged as the strongest associated factor: men with a non-binary identity (while registered as male) showed the highest symptom levels, followed by transgender men. Additional relevant factors associated with higher symptom levels (*β* ≈ 1) included residing in Italian-speaking regions, unemployment, and second-generation or higher migration background. Conversely, being in a partnership and higher educational attainment were associated with lower GAD-7 scores, indicating protective effects.

### 3.3 Depressive symptoms

Among men in the weighted sample, 6.3% (*n* = 224,908; 6,533 missing responses) reported experiencing depressive symptoms within the past 12 months. Of these, 148,633 respondents indicated having received a medical diagnosis of depression, corresponding to a weighted prevalence of 4.2%.

The mean PHQ-9 score was *M* = 3.71 (SD = 4.03; 81,382 missing responses). According to established PHQ-9 cut-off values, 69.2% (*n* = 2,401,829) of men reported no symptoms, 22.8% (*n* = 790,659) reported mild symptoms, 4.9% (*n* = 169,011) reported moderate symptoms, 2.3% (*n* = 79,839) reported moderately severe symptoms, and 0.8% (*n* = 27,667) reported severe symptoms.

For the association analyses, the PHQ-9 total score was used as the dependent variable in multiple linear regression models (see Supplementary Table S2). All sociodemographic variables were significantly associated with depressive symptom levels.

**Figure 2:**
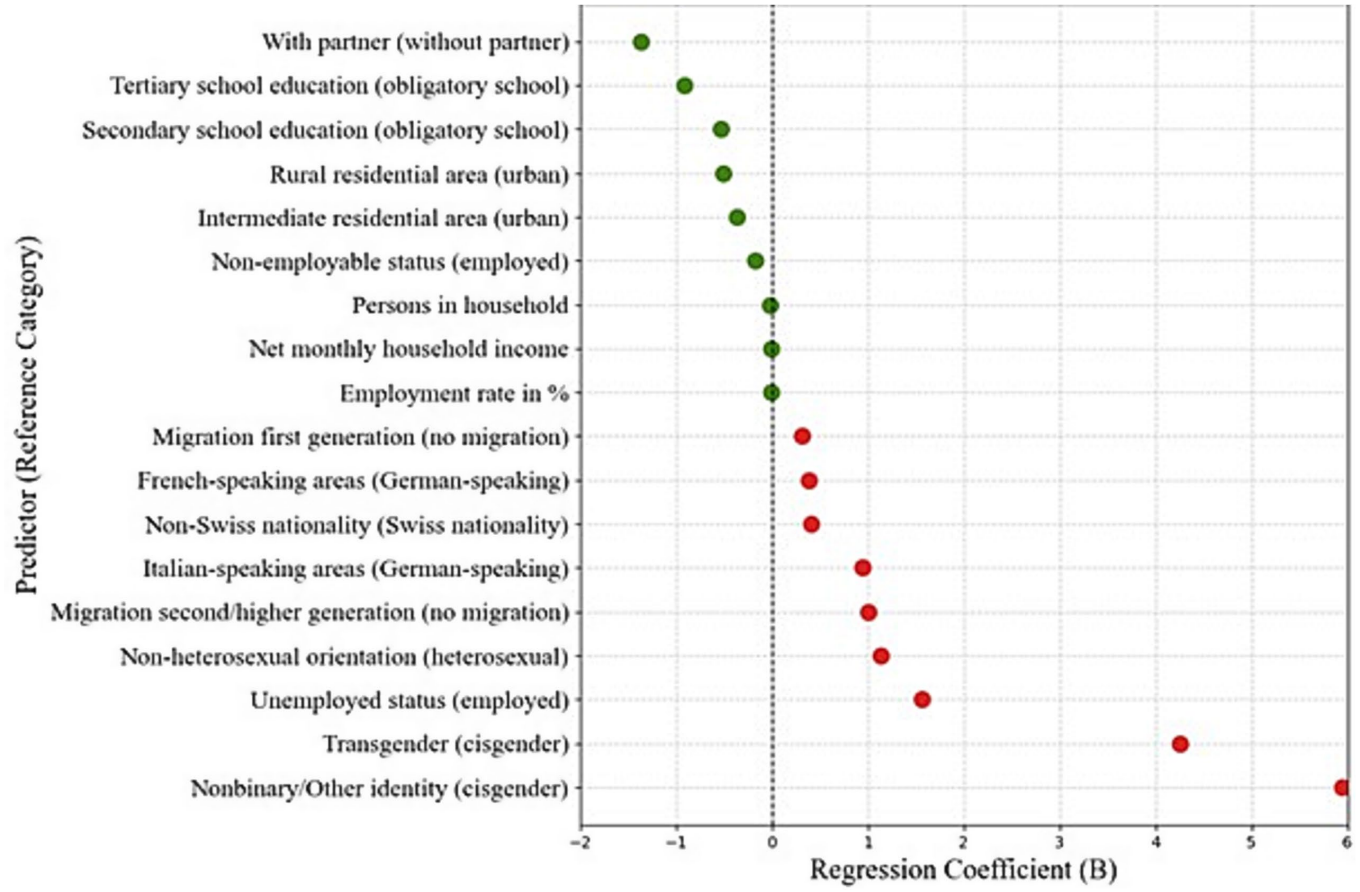
Associated factors of depression (PHQ-9) based on regression coefficients. Green dots represent protective factors and red dots indicate factors associated with higher symptom levels. The 95% confidence intervals are not visible due to the narrow range.

As illustrated in Figure 2, men with a non-binary identity (while registered as male) exhibited the highest PHQ-9 scores, followed by transgender men. Additional relevant factors associated with higher symptom levels (β ≈ 1) included unemployment, non-heterosexual orientation, second- or higher-generation migration background, and residing in Italian-speaking regions. Conversely, being in a partnership and higher educational attainment were associated with lower PHQ-9 scores, suggesting protective effects.

### 3.4 Suicidal behavior

Data on lifetime suicide attempts (11,024 missing responses) indicated that 2.6% (*n* = 91,981) of men reported one suicide attempt, and 0.7% (*n* = 26,463) reported multiple attempts. The vast majority (96.7%) reported no history of suicide attempts.

Among respondents who had attempted suicide, 43.3% (*n* = 51,020) reported having spoken to someone in their private social network about the attempt, whereas 56.6% (*n* = 66,605) had not done so. Regarding contact with healthcare professionals, 56.2% (*n* = 66,102) indicated that they had discussed their attempt with a physician or healthcare provider, while 43.8% (*n* = 51,523) had not. When asked about counseling or institutional support, 92.3% (*n* = 108,584) reported having received support, whereas 7.7% (*n* = 9,041) had not sought such help. Finally, 78.2% (*n* = 91,942) stated that they had not spoken to anyone about their suicide attempt, while 21.8% (*n* = 25,683) reported having shared their experience with someone (without specification).

Binary logistic regression analyses were conducted with lifetime suicide attempt (yes/no) as the dependent variable (see Supplementary Table S3). All examined sociodemographic variables were significantly associated with suicide attempts.

**Figure 3:**
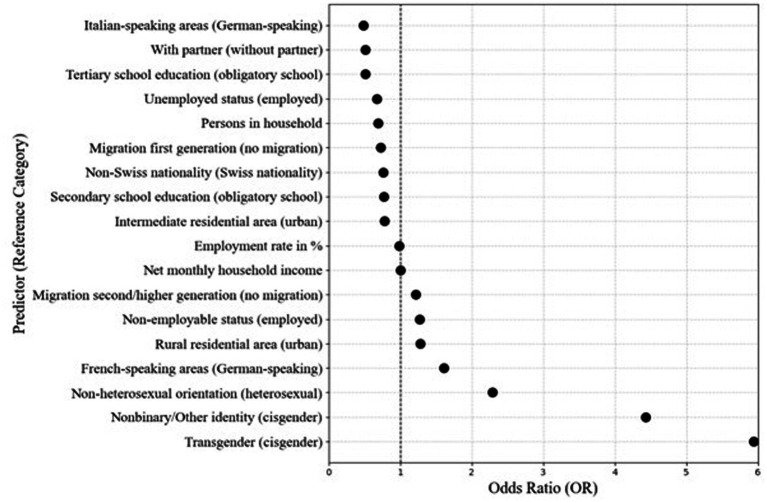
Associated factors of suicide attempts based on odds ratios. The 95% confidence intervals are not visible due to the narrow range.

As illustrated in Figure 3, transgender men had an OR = 5.94, indicating nearly six times higher odds of having attempted suicide compared with cisgender men. Men with non-binary or “other” gender identities showed similarly elevated odds (OR = 4.43). Non-heterosexual men were approximately 2.29 times more likely to have attempted suicide than heterosexual men. Regional differences were also observed: men living in German-speaking regions showed higher odds relative to those in Italian-speaking regions (OR = 2.04), while men in French-speaking areas had 1.61 times higher odds than those in German-speaking regions. Additionally, not being in a partnership and lower educational attainment were associated with increased odds of lifetime suicide attempts (both OR ≈ 1.96).

## 4 Discussion

### 4.1 Sociodemographic and cultural risk profiles in men’s mental health

Using nationally representative data from the SHS 2022, this study examined the prevalence and correlates of generalized anxiety, depression, and suicide attempts among men in Switzerland. The findings reveal distinct yet partially overlapping vulnerability patterns shaped by demographic, socioeconomic, and psychosocial factors, underscoring the heterogeneity of mental health burden within male populations.

Gender-diverse individuals registered as male in civil status records showed the highest overall psychological burden. Men with non-binary or “other” gender identities showed consistently higher symptom levels and higher odds across all outcomes, while transgender men had the highest odds of suicide attempts and comparably high symptom levels for depression and anxiety. These findings indicate that gender identity—a social construct reflecting one’s internal sense of self (Hambruch et al., 2025)—is closely associated with mental-health disparities among men. These disparities can be interpreted in light of masculinities theories, which highlight how deviation from hegemonic masculine norms is socially sanctioned and associated with increased psychosocial vulnerability. This pattern is consistent with international evidence documenting substantially higher rates of depression, anxiety, and suicidality among gender minorities compared with cisgender individuals (Chakraborty et al., 2011; Hirsch et al., 2017; Burns et al., 2018; Borgogna et al., 2019; Guz et al., 2021). Our results extend this literature by demonstrating that such disparities are also evident in a nationally representative Swiss sample of men.

Similarly, men identifying as non-heterosexual displayed higher symptom levels and higher odds across depression, anxiety, and suicidality. This finding underscore persistent mental health inequities among sexual and gender minorities (SGM) and aligns with previous population-based evidence from Switzerland and other Western countries showing increased prevalence of mental health problems among sexual minority men (Wang et al., 2007b, 2014; Malik et al., 2023). These disparities are commonly explained through minority stress processes, including stigma, discrimination, and social exclusion, which may exacerbate psychological vulnerability (Courtenay, 2000). In addition, divergence from hegemonic, heteronormative masculinity ideals may further contribute to distress among SGM men, positioning sexual minority men in subordinated or marginalized masculinity categories within gender hierarchies (Connell and Messerschmidt, 2005; Messerschmidt, 2019).

Masculinity norms, transmitted through family, education, and media (Inckle, 2014; Affleck et al., 2018; Rice et al., 2018), shape emotional expression, coping, and health-related behaviors. Symptoms such as irritability, agitation, or risk-taking—frequently observed in men—do not always align with conventional diagnostic criteria for depression or anxiety (Möller-Leimkühler, 2003; Addis, 2008; Bilsker et al., 2018; Walther, 2025). As a result, some men externalize, minimize or deny psychological distress (Seidler et al., 2016; Rice et al., 2018), reframing symptoms in ways that remain compatible with dominant masculinity norms. Recent conceptual work further suggests that male-typical expressions of distress may contribute to systematic underrecognition and underdiagnosis of mental health problems among men (Walther, 2025).

Research has consistently shown that traditional masculine role norms hinder help-seeking among men (Möller-Leimkühler, 2002; Seidler et al., 2016). While help-seeking behaviors are relevant for understanding underrecognition and delayed care, they were not directly examined in the present study and are therefore discussed here solely as a contextual framework for interpreting prevalence patterns. Seeking professional help typically involves multiple stages, ranging from symptom recognition to the decision to seek care (McLaren et al., 2023; Hammer et al., 2024). However, many men avoid professional assistance because they associate it with weakness or femininity (Vandello and Bosson, 2013). Instead, they may rely on coping strategies congruent with traditional masculinity norms, such as alcohol use, emotional suppression, or excessive self-reliance (Spendelow, 2015; Whittle et al., 2015), which may partly explain why comorbidity of depression and anxiety with substance-use disorders is more common among men than among women (Bolton et al., 2009). In line with this research, population-based studies from Turkey indicate that men are less likely than women to seek psychological support, even when mental health needs are evidently present (Güney et al., 2024; Ercan et al., 2025). These findings provide illustrative evidence for gendered help-seeking patterns, while reflecting downstream service-utilization processes within a specific cultural and healthcare context.

Beyond gender-related factors, structural and socioeconomic factors emerged as salient associated factors of men’s mental health. Unemployment was associated with higher levels of depression and anxiety, whereas being non-employable (e.g., retired or permanently disabled) was linked to increased odds of suicide attempts. Interestingly, unemployment itself was not associated with higher suicide risk, potentially reflecting buffering mechanisms such as Switzerland’s social-welfare system or cultural differences in stigma surrounding job loss. Although unemployment is generally associated with elevated suicide risk (Milner et al., 2014), this relationship often attenuates when socioeconomic covariates are taken into account (Fergusson et al., 2007). The duration and subjective meaning of unemployment—factors not captured in the present data—may further modulate risk (Milner et al., 2013). Given that achievement and productivity are central components of hegemonic masculine identity, prolonged exclusion from the labor market may be particularly distressing for men who strongly endorse conventional masculinity norms (Milner et al., 2013; Rice et al., 2018, 2024).

Systemic biases in healthcare may further compound this vulnerability. Previous research suggests that physicians spend less time with male patients and are less likely to inquire about emotional distress (Courtenay, 2000; Brooks, 2001; Möller-Leimkühler, 2002; Dey and Jorm, 2017; Walther, 2025), potentially contributing to underdiagnosis and delayed treatment among men (Borowsky et al., 2000). Such mechanisms may reinforce observed prevalence patterns and contribute to unmet mental health needs in male populations.

A Switzerland-specific pattern also emerged with respect to language region. Men from Italian-speaking regions reported moderately higher anxiety and depressive symptoms, whereas those from French-speaking regions showed the highest prevalence of suicide attempts compared with German-speaking men. These findings are consistent with evidence of micro-cultural differences across Swiss language regions in stress perception, emotion expression, and responses to loss of autonomy in illness contexts (Schulz et al., 2013; Roser et al., 2019).

Beyond linguistic variation, Swiss language regions also differ in aspects such as socioeconomic structures and health-system organization. Previous research has documented region-specific differences in morbidity profiles, access to ambulatory and inpatient care, and overall patterns of healthcare use, which may shape differential exposure to stressors as well as opportunities for early detection and support (Busato and Künzi, 2008; Bähler et al., 2020). Such contextual characteristics may contribute to regional vulnerability or protection regarding men’s mental health, without implying direct causal mechanisms. Regional characteristics therefore appear to represent an important contextual factor in men’s mental health and warrant consideration in both epidemiological research and intervention planning.

Migration background also played a role. Men with second- or higher-generation migration status showed higher symptom levels and higher odds for depression and anxiety but only slightly higher odds of suicide attempts. This pattern aligns with evidence suggesting that second-generation migrants often experience higher levels of discrimination and weaker community ties than first-generation migrants, whose migration is frequently selective for health and resilience (Mindlis and Boffetta, 2017; Kiselev et al., 2020). Over time, this initial health advantage may diminish, underscoring the importance of preventive efforts tailored to later-generation migrant populations.

Some associated factors showed limited practical relevance despite statistical significance. The number of household members displayed inconsistent associations, and household income, although statistically significant due to the large sample size, showed only minimal effect sizes across outcomes, suggesting limited clinical importance.

Conversely, several factors appeared protective across outcomes. Being in a relationship was consistently associated with lower levels of anxiety and depression and reduced odds of suicide attempts, highlighting the buffering role of emotional and social support. From a positive masculinity perspective, relational commitment and social responsibility may represent socially valued masculine practices that function as protective resources rather than sources of strain (Kiselica and Englar-Carlson, 2010).

Higher educational attainment was also linked to more favorable mental-health outcomes. Moreover, living in intermediate or rural areas was associated with fewer depressive symptoms and suicide attempts, although rural residence was slightly related to higher anxiety levels. These findings are consistent with mixed international evidence on urban–rural mental health differences (Weeks et al., 2004; Probst et al., 2006; Peen et al., 2010; Bonnell et al., 2022) and point to the contextual complexity of geographic influences.

Taken together, these results underscore that masculinity is multifaceted and context-dependent, shaped by intersecting social, cultural, and structural forces. Gender and sexual identity remain underassessed in population health research (Bragazzi et al., 2022; Hambruch et al., 2025). By including both cisgender and gender-diverse men in a large representative sample, the present study contributes to closing that gap and advances understanding of mental health disparities within diverse male populations.

Importantly, these relative differences within male populations should not be misinterpreted as indicating low levels of psychological distress among men who are more closely aligned with hegemonic masculinity norms. International evidence consistently demonstrates that men, despite reporting lower prevalence of depression and anxiety than women, show substantially higher suicide rates and greater engagement in externalizing behaviors, including substance use and risk-taking (Shah et al., 2007; Nock et al., 2008; Seedat et al., 2009). This observation has been documented across a wide range of cultural contexts and underscores that mental health vulnerability among men may be expressed in forms that are less likely to be captured by symptom-based screening instruments (Oliffe et al., 2020; River and Flood, 2021). In this sense, closer alignment with hegemonic masculinities may be associated with relative advantages in social legitimacy and reduced minority stress, while simultaneously reinforcing dysfunctional norms of emotional restraint and self-reliance that delay help-seeking and contribute to elevated suicide risk. Comparisons with women in the studies mentioned above further highlight that lower symptom prevalence among men does not equate to lower overall mental health burden, but rather reflects gendered patterns of distress expression, recognition, and response within healthcare systems.

### 4.2 Limitations and future directions

Several limitations should be considered when interpreting these findings. First, the study relied on self-reported measures, which may introduce response bias or underreporting of sensitive information such as mental health symptoms or suicidal behavior. Although the PHQ-9 and GAD-7 are validated instruments with strong psychometric properties, they are screening tools rather than diagnostic assessments. Second, the cross-sectional design precludes causal inferences, and observed associations should therefore be interpreted as correlational. Third, despite rigorous weighting and quality control, nonresponse and sampling biases may persist, particularly among hard-to-reach populations such as men with severe mental illness or marginalized gender identities. Fourth, the study did not include direct measures of masculinity norms, gender role conflict, or minority stress, which may help explain observed disparities.

In addition, although standard assumptions of regression analyses were considered in the present study, formal diagnostic tests were limited. Independence of observations is ensured by the individual-level sampling design of the SHS, and multicollinearity among associated factors was empirically assessed and found to be negligible. Other assumptions of the logistic regression, such as linearity of the logit for continuous factors and the influence of individual outliers, were not formally tested. However, given the large, weighted population-based sample and the predominantly categorical specification of factors, potential violations are unlikely to meaningfully affect the reported associations.

Future research should therefore move beyond symptom-based assessments and incorporate contextual and cultural indicators, including beliefs about masculinity, emotional expression, and help-seeking behavior. Evidence consistently links stronger endorsement of traditional masculinity norms with reduced emotional openness and lower help-seeking (Möller-Leimkühler, 2002; Seidler et al., 2016; Rice et al., 2018). Integrating such constructs into large-scale health surveys would enable a more nuanced understanding of how gender socialization shapes men’s mental health and would inform the development of gender-sensitive prevention and intervention strategies.

## 5 Conclusion

This study provides the first population-based overview of men’s mental health in Switzerland, identifying consistent sociodemographic and cultural association patterns across depression, anxiety, and suicidality. Language region, employment status, gender identity, and migration background emerged as central associated factors, while higher education and being in a partnership were consistently protective. Although most associations were moderate in magnitude, they highlight the multifactorial nature of men’s mental health and the importance of addressing both individual- and structural-level factors.

Traditional masculinity norms and gender socialization may obscure the true prevalence and expression of psychological distress among men, contributing to underdiagnosis and delayed support. Addressing these cultural and behavioral dimensions is essential for developing inclusive prevention and intervention strategies that reflect the diversity of men’s experiences. Future research and policy efforts should therefore integrate gender-sensitive and intersectional frameworks to improve early detection, support, and treatment for men at risk of mental illness or suicidality.

Overall, these findings provide an empirical basis for targeted public health initiatives and inform the development of gender-sensitive mental health policies in Switzerland.

## Data Availability

Access to the Swiss Health Survey 2022 dataset is restricted. The anonymized microdata are available only upon request from the Swiss Federal Statistical Office for research purposes under a data use agreement. Public sharing of individual-level data is not permitted in accordance with Swiss data protection laws. Requests to access these datasets should be directed to the Swiss Federal Statistical Office, Neuchâtel, Switzerland. Data access information is available at: https://www.bfs.admin.ch/bfs/en/home.html. Any further inquiries can be directed to the corresponding author.
