# Supplementary material for: Associated factors of depression, anxiety, and suicide behavior among men in Switzerland: findings from the Swiss health survey 2022
Source: Front Psychol. 2026 Feb 4;17:1725181. doi: 10.3389/fpsyg.2026.1725181 (PMC12913440; doi:10.3389/fpsyg.2026.1725181)
Supplement: Supplementary file 1 [file Table_1.docx]

**Supplementary table S1**. Predictors of symptoms of generalized anxiety disorder (GAD).

| **Predictor (reference category)** | **Regression coefficient (B)** | **SE** | **Standardized Coefficient (Beta)** | **T** | **p** | **95% Confidence Interval for B** |
| --- | --- | --- | --- | --- | --- | --- |
| Transgender (cisgender) | 3.88 | 0.03 | 0.08 | 141.48 | <.001 | [3.83; 3.94] |
| Nonbinary/“Other” identity^1^ (cisgender) | 4.66 | 0.04 | 0.06 | 110.63 | <.001 | [4.58; 4.74] |
| Non-heterosexual orientation^2^ (heterosexual) | 0.47 | 0.01 | 0.04 | 66.53 | <.001 | [0.46; 0.49] |
| With partner^3^ (without partner^4^) | -0.73 | <0.01 | -0.11 | -202.48 | <.001 | [-0.74; -0.73] |
| Persons in household | 0.07 | <0.01 | 0.03 | 54.22 | <.001 | [0.07; 0.08] |
| Non-Swiss nationality (Swiss nationality) | 0.47 | <0.01 | 0.06 | 114.02 | <.001 | [0.46; 0.48] |
| Migration first generation (no migration) | 0.38 | <0.01 | 0.05 | 92.81 | <.001 | [0.37; 0.39] |
| Migration second/higher generation (no migration) | 0.91 | 0.01 | 0.08 | 137.48 | <.001 | [0.90; 0.92] |
| Secondary school education (obligatory school) | -0.36 | .01 | -0.05 | -62.99 | <.001 | [-0.37; -0.35] |
| Tertiary school education (obligatory school) | -0.57 | .01 | -0.08 | -100.37 | <.001 | [-0.58; -0.56] |
| Non-employable status (employed) | -0.38 | <0.01 | -0.05 | -90.59 | <.001 | [-0.38; -0.37] |
| Unemployed status (employed) | 1.25 | 0.01 | 0.05 | 100.99 | <.001 | [1.12; 1.28] |
| Employment rate in % | <0.01 | <0.01 | 0.01 | 13.64 | <.001 | [<0.01; <0.01] |
| Net monthly household income^5^ | <-0.01 | <0.01 | <0.01 | -26.18 | <.001 | [<0.01; <0.01] |
| Intermediate residential area (urban) | 0.06 | 0.01 | 0.01 | 10.17 | <.001 | [0.05; 0.07] |
| Rural residential area (urban) | 0.19 | 0.01 | 0.03 | 37.95 | <.001 | [0.18; 0.20] |
| French-speaking areas (German-speaking) | 0.52 | <0.01 | 0.07 | 120.58 | <.001 | [0.51; 0.53] |
| Italian-speaking areas Language areas (German-speaking) | 1.42 | 0.01 | 0.08 | 152.19 | <.001 | [1.40; 1.44] |

*Note. ^1^Individuals registered as male in civil status records but who have a non-binary (or “other,” without specification) gender identity. ^2^For this analysis, the categories gay, bisexual, and “other” (without specification) were merged to non-heterosexual. ^3^Single = single, widowed, divorced, unmarried, dissolved registered partnership. ^4^With partner = married, registered partnership. ^5^ income in CHF.*
