# Supplementary material for: Associated factors of depression, anxiety, and suicide behavior among men in Switzerland: findings from the Swiss health survey 2022
Source: Front Psychol. 2026 Feb 4;17:1725181. doi: 10.3389/fpsyg.2026.1725181 (PMC12913440; doi:10.3389/fpsyg.2026.1725181)
Supplement: Supplementary file 2 [file Table_2.docx]

**Supplementary table S2**. Predictors of symptoms of depression (PHQ-9).

| **Predictor (reference category)** | **Regression coefficient (B)** | **SE** | **Standardized Coefficient (Beta)** | **T** | **p** | **95% Confidence Interval for B** |
| --- | --- | --- | --- | --- | --- | --- |
| Transgender (cisgender) | 4.26 | 0.03 | 0.70 | 128.86 | <.001 | [4.20; 4.33] |
| Nonbinary/“Other” identity^1^ (cisgender) | 5.95 | 0.05 | 0.06 | 115.07 | <.001 | [5.85; 6.06] |
| Non-heterosexual orientation^2^ (heterosexual) | 1.13 | 0.01 | 0.07 | 133.67 | <.001 | [1.11; 1.14] |
| With partner^3^ (without partner^4^) | -1.36 | <0.01 | -0.17 | -318.53 | <.001 | [-1.37; -1.35] |
| Persons in household | -0.02 | <0.01 | -0.01 | -9.71 | <.001 | [-0.02; -0.01] |
| Non-Swiss nationality (Swiss nationality) | 0.41 | 0.01 | 0.05 | 83.12 | <.001 | [0.40; 0.42] |
| Migration first generation (no migration) | 0.31 | 0.01 | 0.04 | 63.51 | <.001 | [0.30; 0.32] |
| Migration second/higher generation (no migration) | 1.01 | 0.01 | 0.07 | 128.52 | <.001 | [0.99; 1.02] |
| Secondary school education (obligatory school) | -0.53 | 0.01 | -0.06 | -77.76 | <.001 | [-0.54; -0.51] |
| Tertiary school education (obligatory school) | -0.91 | 0.01 | -0.11 | -136.45 | <.001 | [-0.92; -0.90] |
| Non-employable status (employed) | -0.17 | 0.01 | -0.02 | -33.35 | <.001 | [-0.18; -0.16] |
| Unemployed status (employed) | 1.56 | 0.02 | 0.06 | 106.76 | <.001 | [1.53; 1.59] |
| Employment rate in % | <-0.01 | <0.01 | -0.01 | -19.36 | <.001 | [<-0.01; <0.01] |
| Net monthly household income^5^ | <-0.01 | <0.01 | -0.03 | -33.76 | <.001 | [<0.01; <0.01] |
| Intermediate residential area (urban) | -0.36 | 0.01 | -0.04 | -66.63 | <.001 | [-0.37; -0.35] |
| Rural residential area (urban) | -0.51 | 0.01 | -0.05 | -84.74 | <.001 | [-0.53; -0.50] |
| French-speaking areas (German-speaking) | 0.39 | 0.01 | 0.04 | 76.50 | <.001 | [0.38; 0.40] |
| Italian-speaking areas Language areas (German-speaking) | 0.95 | 0.01 | 0.05 | 85.22 | <.001 | [0.93; 0.97] |

*Note. ^1^Individuals registered as male in civil status records but who have a non-binary (or “other,” without specification) gender identity. ^2^For this analysis, the categories gay, bisexual, and “other” (without specification) were merged to non-heterosexual. ^3^Single = single, widowed, divorced, unmarried, dissolved registered partnership. ^4^With partner = married, registered partnership. ^5^ income in CHF.*
