# Supplementary material for: Associated factors of depression, anxiety, and suicide behavior among men in Switzerland: findings from the Swiss health survey 2022
Source: Front Psychol. 2026 Feb 4;17:1725181. doi: 10.3389/fpsyg.2026.1725181 (PMC12913440; doi:10.3389/fpsyg.2026.1725181)
Supplement: Supplementary file 3 [file Table_3.docx]

**Supplementary table S3**. Predictors of suicide attempts.

| **Predictor (reference category)** | **Regression coefficient (B)** | **SE** | **Exp (B)** | **Wald** | **p** | **95% Confidence Interval Exp (B)** |
| --- | --- | --- | --- | --- | --- | --- |
| Transgender (cisgender) | 1.78 | 0.02 | 5.94 | 7560.97 | <.001 | [5.71; 6.19] |
| Nonbinary/“Other” identity^1^ (cisgender) | 1.41 | 0.04 | 4.43 | 1357.40 | <.001 | [3.81; 4.43] |
| Non-heterosexual orientation^2^ (heterosexual) | 0.83 | 0.01 | 2.29 | 9090.12 | <.001 | [2.25; 2.33] |
| With partner^3^ (without partner^4^) | -0.68 | 0.01 | 0.51 | 12087.59 | <.001 | [0.50; 0.51] |
| Persons in household | -0-37 | <0.01 | 0.69 | 18530.72 | <.001 | [0.69; 0.70] |
| Non-Swiss nationality (Swiss nationality) | -0.28 | 0.01 | 0.76 | 1485.66 | <.001 | [0.75; 0.77] |
| Migration first generation (no migration) | -0.31 | 0.01 | 0.73 | 1888.42 | <.001 | [0.72; 0.74] |
| Migration second/higher generation (no migration) | 0.20 | 0.01 | 1.22 | 411.17 | <.001 | [1.20; 1.24] |
| Secondary school education (obligatory school) | -0.23 | 0.01 | 0.77 | 789.29 | <.001 | [0.78; 0.81] |
| Tertiary school education (obligatory school) | -0.67 | 0.01 | 0.51 | 6162.11 | <.001 | [0.50; 0.52] |
| Non-employable status (employed) | 0.24 | 0.01 | 1.27 | 1403.40 | <.001 | [1.26; 1.29] |
| Unemployed status (employed) | -0.41 | 0.03 | 0.67 | 258.80 | <.001 | [0.64; 0.70] |
| Employment rate in % | -0.01 | <0.01 | 0.99 | 2176.44 | <.001 | [0.99; 0.99] |
| Net monthly household income^5^ | <0.01 | <0.01 | 1.00 | 4813.44 | <.001 | [1.00; 1.00] |
| Intermediate residential area (urban) | -0.26 | 0.01 | 0.78 | 1023.84 | <.001 | [0.76; 0.79] |
| Rural residential area (urban) | 0.25 | 0.01 | 1.28 | 1065.26 | <.001 | [1.26; 1.30] |
| French-speaking areas (German-speaking) | 0.48 | 0.01 | 1.61 | 5660.17 | <.001 | [1.59; 1.63] |
| Italian-speaking areas Language areas (German-speaking) | -0.71 | 0.02 | 0.49 | 1042.65 | <.001 | [0.47; 0.51] |

*Note. ^1^Individuals registered as male in civil status records but who have a non-binary (or “other,” without specification) gender identity. ^2^For this analysis, the categories gay, bisexual, and “other” (without specification) were merged to non-heterosexual. ^3^Single = single, widowed, divorced, unmarried, dissolved registered partnership. ^4^With partner = married, registered partnership. ^5^ income in CHF.*
